# Integration of bulk and single-cell RNA-seq reveals prognostic gene signatures in patients with bladder cancer treated with immune checkpoint inhibitors

**DOI:** 10.1007/s00262-024-03839-7

**Published:** 2024-12-21

**Authors:** Mina Cho, Hyun Chang, Ju Han Kim

**Affiliations:** 1 Division of Biomedical Informatics, Seoul National University Biomedical Informatics (SNUBI), Seoul National University College of Medicine, Seoul, 03080 Republic of Korea; 2 Medical Oncology and Hematology, College of Medicine, International St Mary’s Hospital, Catholic Kwandong University, Incheon, 22711 Republic of Korea

**Keywords:** Bladder cancer, Bladder cancer gene signature, Immune checkpoint inhibitors, Tumor microenvironment, Tumor mutation burden

## Abstract

Immune checkpoint inhibitors have significantly advanced research in oncology and are used to successfully treat patients with bladder cancer (BC). However, as the benefits of programmed death-1/ programmed death-ligand-1 blockade immunotherapy do not extend to all patients with BC, biomarkers are required to improve prognostic stratification. This study aims to identify reliable biomarkers to enhance the prediction of treatment outcomes. Bulk RNA expression data from a BC cohort (GSE176307) receiving ICI and single-cell sequencing data from patients with BC (GSE135337) were collected. We identified differentially expressed genes (DEGs) within cells that were associated with favorable survival outcomes and developed a predictive bladder cancer gene signature (BC-GS). Subsequently, we performed pathway enrichment analysis using the Reactome database. We used two independent datasets to validate the BC-GS. Patients with low BC-GS had a significantly shorter overall survival (OS) than those with high BC-GS (*p* < 0.05, *p* < 0.001, respectively). Additionally, patients with a concurrently low BC-GS score and low tumor mutation burden (TMB) in GSE176307 and the two validation datasets exhibited an increased risk of death. Genes in the BC-GS were predominantly involved in CD8^+^ T cell activation, antigen presentation, and immune checkpoint pathways. CIBERSORT analysis revealed differences in CD4^+^ T cells and macrophages between the high and low BC-GS groups. This study demonstrated the prognostic significance of the BC-GS in patients with BC treated with ICI. The combined assessment of the BC-GS and TMB may provide a sophisticated prognostic approach to enhance patient stratification for ICI treatment in BC.

**Supplementary Information:**

The online version contains supplementary material available at 10.1007/s00262-024-03839-7.

## Introduction

Bladder cancer (BC) is the most prevalent cancer affecting the urinary system that poses significant therapeutic challenges, especially in the advanced stages of the disease [1, 2]. The effectiveness of conventional treatments in improving outcomes for patients with locally advanced or metastatic bladder cancer is often limited. The introduction of immune checkpoint inhibitors (ICIs) targeting the programmed death-1 (PD-1)/ PD-ligand-1 (PD-L1) pathways has revolutionized the treatment landscape by significantly improving survival rates for these patients [2–6]. ICIs are effective in various treatment scenarios, such as in combination with chemotherapy as the first line of treatment, treating patients unable to receive platinum-based therapies with pembrolizumab, maintenance therapy with avelumab, and in monotherapy as the second line of treatment [3–7]. However, patient responses to these therapies can vary considerably, underscoring the importance of developing reliable predictive biomarkers to guide treatment decisions. This necessitates a deeper understanding of the heterogeneous nature of BC and the intricate dynamics of the tumor microenvironment (TME). Despite these urgent requirements, robust biomarkers that can predict patient response to ICIs have not been identified or validated.

Currently, a combined approach that utilizes scRNA-seq and RNA-seq is used to enhance understanding regarding tumor heterogeneity [8, 9]. This method can improve the identification of differentially expressed genes (DEGs), thereby refining tumor heterogeneity analysis and prognostic modeling. It also enables a more detailed analysis of the TME dynamics. Robust predictive biomarkers that can considerably enhance patient stratification and optimize treatment outcomes in BC can be established by combining these methodologies.

In this study, we aimed to integrate scRNA-seq and bulk RNA-seq expression data to identify prognostic biomarkers for patients with BC undergoing ICI therapy as a palliative treatment (Supplementary Fig. 1). We developed a prognostic biomarker, the bladder cancer gene signature (BC-GS), which was validated in two independent datasets, and demonstrated its strong discriminative ability. The genes in the BC-GS were predominantly expressed in pathways associated with immune responses, particularly those involving CD8^+^ T cell activation, antigen presentation, and immune checkpoint pathways. CIBERSORT analysis revealed differences in CD4^+^ T cells and macrophages between the high and low BC-GS groups. These results indicated that the BC-GS may be used to predict survival outcomes in patients with advanced BC undergoing treatment with ICIs.

## Materials and methods

### Data collection

The RNA-seq expression data of patients with BC were obtained from three immunotherapy cohorts: IMvigor210, UC-GENOME, and GSE176307. IMvigor210 is a single-arm phase II study that investigated the effects of atezolizumab in patients with metastatic BC [10]. UC-GENOME is a genome analysis and biospecimen repository study of patients with metastatic BC [11]. Finally, GSE176307 is a retrospective study of patients with advanced BC who received ICI treatment at a single academic medical center [12].

The scRNA-seq data were obtained from GSE135337, which consisted of seven patients with BC [13]. In this study, droplet-based scRNA-seq was used to determine the transcriptional profiles of these cells. Detailed information regarding these datasets is provided in Supplementary Table 1.

### Clinical and RNA-seq data

Clinical and bulk RNA-seq data from GSE176307 included 89 BC samples from an original cohort of 103 patients. Transcripts per million (TPM) values were used as input for the Scissor method [8] after removing duplicate samples from the dataset. These TPM values were obtained by aligning reads to GrCH38 (v22) using the STAR/Salmon pipeline and quantifying expression using RSEM [8]. RNA-seq data and clinical information from IMvigor210 (*n* = 348) were obtained using the R package IMvigor210CoreBiologies [10]. TPM from RNA-seq data of IMvigor210 was used. A subset of 195 patients from the original cohort of 348 patients in the IMvigor210 study was selected based on the origin of the bladder tissue. Gene expression matrix and clinical information of the UC-GENOME (*n* = 180) were deposited in the cBioPortal database under the study titled “Urothelial Carcinoma, Nature Communications (BCAN/HCRN 2022)” and were downloaded using the R package cgdsr (v1.3.0) [11, 14]. Among the original cohort of 180 patients from the UC-GENOME study, a subset of 101 patients was selected for further analysis, considering the availability of data on immunotherapy treatment status, gene expression, and survival (Supplementary Fig. 2). The UC-GENOME dataset exclusively offers log2-normalized z-scores that have been processed using upper quantile normalization; these data were used in the present study (Supplementary Table 1).

Quality control for the scRNA-seq data of GSE135337 was performed by filtering cells with reduced gene expression in each dataset using specific parameters outlined in Supplementary Table 2. After the quality control, the data were normalized using the “SCTransform” method [15]. Cluster analysis was performed using the first 30 principal components with a resolution of 1.0. The resolution values were heuristically optimized based on the silhouette score [16, 17]. The clusters were annotated using well-established marker genes for each cell type, such as myeloids/macrophages, T cells, B cells, fibroblasts, and endothelial cells. Marker genes used for annotation were obtained from Lai et al. (2021) (Supplementary Table 3) [13]. A subset excluding epithelial cells was selected for further analyses. The scRNA-seq data were preprocessed using the Seurat R package (v4.3.0) before using the Scissor method [8, 18].

Tumor mutation burden (TMB) values from the IMvigor210, UC-GENOME, and GSE176307 cohorts were used for subsequent analysis. TMB of IMvigor210 was obtained using whole-exome sequencing (WES), while targeted mutation panels were used for the other cohorts.

### Determining the sub-population associated with overall survival (OS)

A subset of 1,517 cells was curated from a larger pool that included immune cells, fibroblasts, and endothelial cells based on the expression of specific marker genes, as shown in Supplementary Table 3 (Supplementary Fig. 3). This selection was made to better understand the role of the TME in influencing patient outcomes after immunotherapy; epithelial cells were excluded to enhance the outcome of the analysis. Toward this, the Scissor method was used to analyze the pre-processed bulk RNA-seq and scRNA-seq data. The Scissor method was configured with a “Cox” family argument and an alpha value of 0.1, while the default settings were used for the remaining parameters [8].

### Analysis of DEGs

To identify the DEGs in Scissor^−^ cells compared to that in other cells without Scissor^+^ cells, the “FindMarkers” function in Seurat with the DESeq test argument was used [18]. To mitigate bias toward highly expressed genes, pseudobulk methods were used instead of single-cell differential expression approaches [19]. DEGs were identified based on statistical significance at the 0.05 level of the false discovery rate (FDR) and a log2 fold change of expression levels greater than 1.5 between the two groups.

### Gene enrichment and network analysis

To elucidate the biological background of the DEGs, gene enrichment analysis was performed using R packages org.Hs.eg.db (v3.16.0) and ReactomePA (v1.42.0) [20, 21]. Additionally, a network analysis was performed using GeneMANIA with default settings [22].

### BC-GS score

Scissor^⁻^ cells associated with improved survival were specifically used to identify prognostic biomarkers. After determining the DEGs between Scissor^⁻^ cells and other cells without Scissor^+^ cells, 10 stably overexpressed genes (*SSR4, RGS1, HLA-DRB5, APOE, C1QB, C1QA, APOC1, JCHAIN, C1QC*, and *DERL3*) across the datasets were carefully selected for further analysis. The TPM expression values of these 10 genes were subjected to transformation using the “voom” function of the LIMMA R package (v3.54.2) [23] and subsequently converted to z-scores in independent bladder cancer datasets (GSE176307 and IMvigor210). Since we had a z-scored gene expression matrix in UC-GENOME, these values were directly used for the analysis. The BC-GS score was calculated as the average of the z-scores of the 10 selected genes.

### Cancer biology-related pathway profiles

To evaluate the variation in gene expression profiles within the essential pathways related to the BC-GS and TMB, the expression levels of genes within these cancer biology-related pathways were assessed, as defined by Mariathasan et al. (2018) (Supplementary Table 4) [10]. These pathways included various processes, such as growth factor receptor signaling, T cell-mediated immunity, antigen presentation mechanism (APM), cell cycle dynamics, tumor growth factor (TGF-*β*) signaling in fibroblasts, angiogenic activity, and epithelial–mesenchymal transition. The GSE176307 target cohort, from which the BC-GS was derived, was analyzed, as distinct survival curves were obtained after using the combination of BC-GS and TMB (Fig. 4b). This distinction highlights the differences in gene expression levels, which will be useful for understanding the impact of these biomarkers on core biological pathways.

### CIBERSORT analysis

The RNA-seq data were deconvoluted using the CIBERSORT [24] method in the IOBR (v0.99.8) [25] R package. For cell distribution analysis, CIBERSORT was applied exclusively to the GSE176307 and IMvigor210 datasets, and the UC-GENOME dataset was omitted because of the high incidence of not available (NA) values.

### Statistical and survival analyses

Categorical variables were compared using Fisher’s exact or Chi-square tests, while continuous variables were analyzed using t tests or Wilcoxon rank-sum tests for non-normal distributions. In cases of three or more groups, analysis of variance (ANOVA) was performed, followed by Tukey’s post hoc analysis to elucidate intergroup differences. Boxplots were used to visualize the data, with boxes representing the interquartile range and the midline indicating the median. Violin plots were used to visually represent the data distribution, with the median shown as a black dot. Survival curves were generated using the Kaplan−Meier method. The statistical significance of survival differences between groups was evaluated using the log-rank test with the survival (v3.2.3) [26] and survminer (v0.4.9) [27] R packages. Hazard ratios (HR) with confidence intervals (CI) were estimated using Cox regression analysis. Statistical analyses were performed using R (v4.2.3) [28].

## Results

### Identifying cells associated with favorable OS

**Fig. 1:**
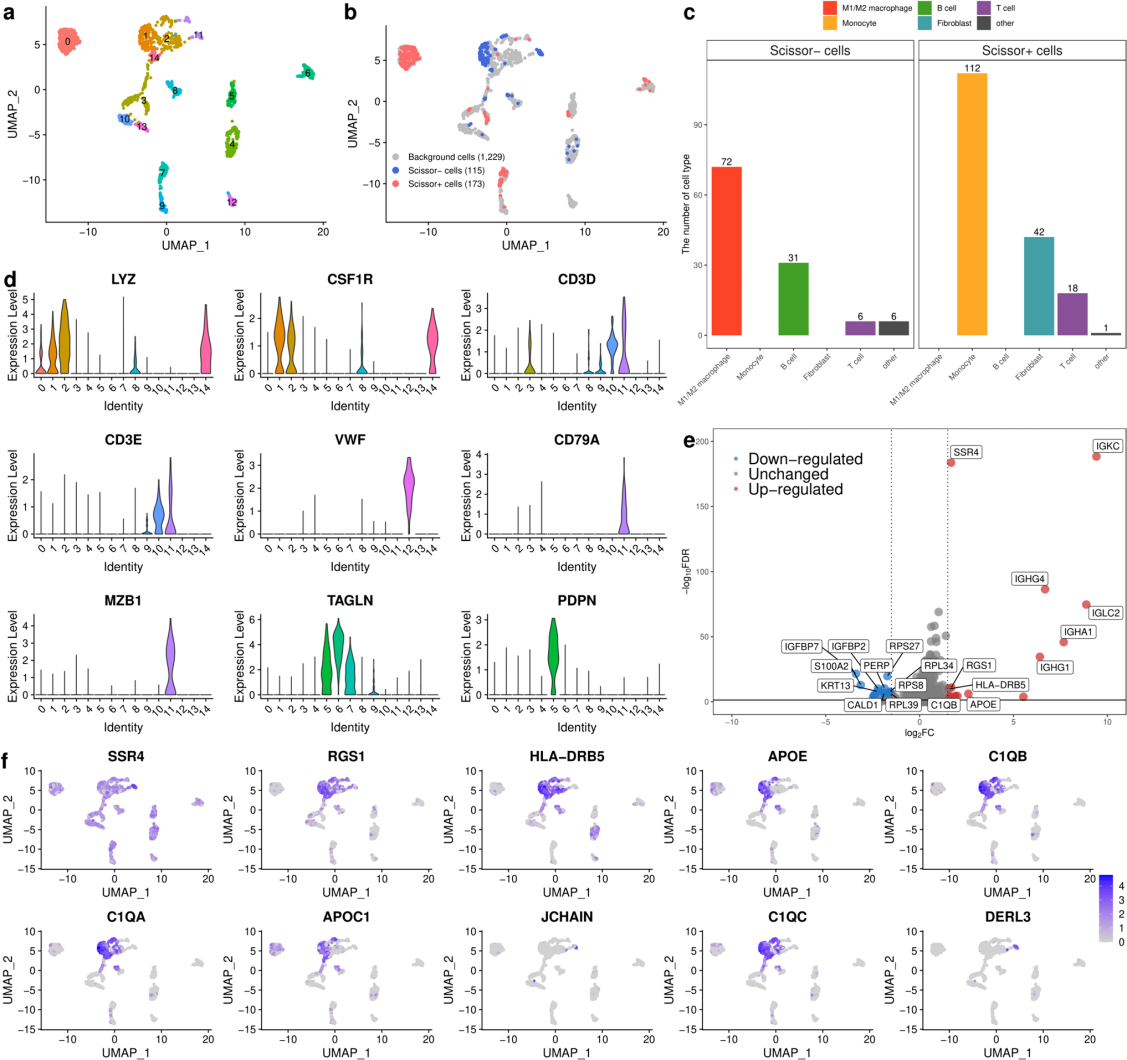
Identification of cells associated with favorable overall survival by integrating single-cell RNA-seq (scRNA-seq) and bulk RNA-seq data. **a** UMAP visualization of 1,517 cells in bladder cancer, except epithelial cells. **b** UMAP visualization of selected cells using the Scissor method. The blue and red dots represent Scissor − (improved survival) and Scissor + cells (poorer survival), respectively. **c** The bar plot shows the cell types associated with Scissor − and Scissor + cells. **d** The violin plot represents the expression of marker genes in 1,517 cells. The cell types included myeloid/macrophages, B cells, T cells, endothelial cells, and fibroblasts. **e** Volcano plot of differential gene expression in Scissor − cells versus all other cells (without Scissor + cells). The two vertical dashed lines represent ± 1.5 log2 fold change of gene expression, and the horizontal dashed line denotes an FDR cutoff of 0.05. The FDR was the adjusted *p*-value calculated using negative binomial generalized linear models. UMAP: uniform manifold approximation and projection

The data of 89 patients with BC in the bulk RNA-seq (GSE176307) dataset and those of seven patients with BC in the scRNA-seq (GSE135337) dataset were analyzed to identify the subpopulation of cells associated with OS (Fig. 1; Supplementary Fig. 1) [12, 13]. We focused on the TME by selecting 1,517 cells from a total of 36,300 cells, including immune cells, fibroblasts, and endothelial cells, and excluding the epithelial cells. Among the 1,517 cells separated into 14 clusters (Fig. 1a), we identified 115 Scissor^⁻^ cells related to improved survival and 173 Scissor^+^ cells related to poor survival (Fig. 1b). The cells associated with improved survival mainly originated from clusters 1 and 11, whereas the cells associated with poor survival predominantly originated from clusters 0, 3, and 7 (Fig. 1a, b). Cells related to improved survival predominantly consisted of M1 or M2 macrophages (63%) and plasma B cells (27%), whereas cells related to poor survival primarily consisted of monocytes (65%) and fibroblasts (24%) (Fig. 1c; Supplementary Fig. 4). The classifications were further refined using additional clustering. Re-clustering improved cell categorization by dividing cluster 11 into plasma B cells or T cells and myeloid/macrophage cells into M1/M2 macrophages or monocytes, which are linked to improved or poor survival, respectively (Fig. 1d; Supplementary Fig. 5, 6, and 7) [29–31]. To elucidate the transcriptional patterns underlying improved survival, we compared the gene expression in these cells with those in other cells without Scissor^+^ cells and identified 15 upregulated and 27 downregulated genes.

### Investigating the clinical and biological significance of BC-GS

**Fig. 2:**
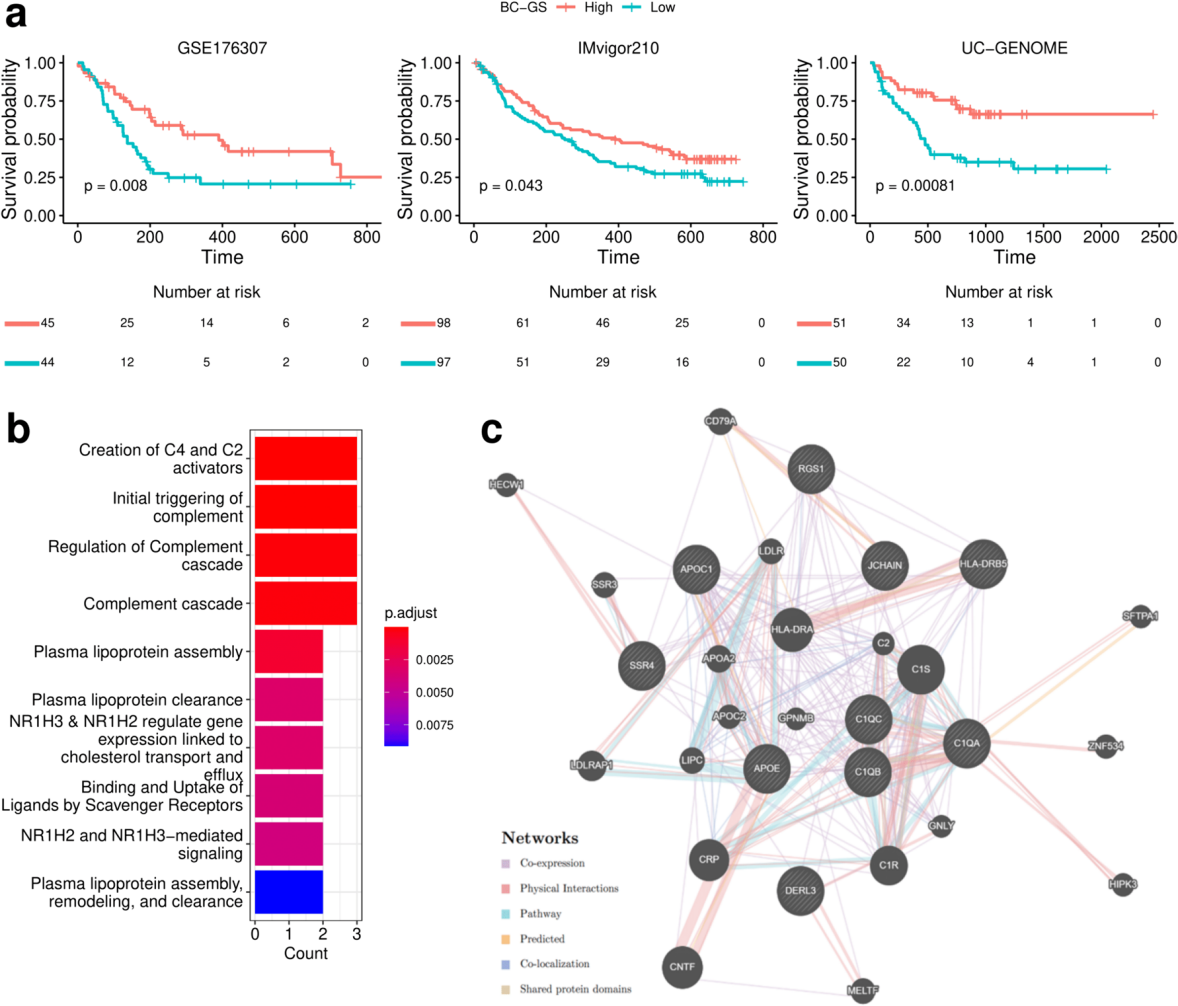
Survival plots according to the BC-GS and pathway enrichment analysis of differentially expressed genes (DEGs). **a** Overall survival (OS) curves show the clinical relevance of bladder cancer gene signatures (BC-GS) in three independent datasets (GSE176307, IMvigor210, and UC-GENOME). Tick marks indicate censoring events. The statistical p-value was determined using the two-tailed log-rank-sum test. **b** Enrichment bar plot of gene signatures in Reactome pathways. **c** Network graph of 10 genes (SSR4, RGS1, HLA-DRB5, APOE, C1QB, C1QA, APOC1, JCHAIN, C1QC, and DERL3) using GENMANIA. The shaded nodes enclosed in the circle represent the 10 genes that were included as inputs

The BC-GS, a prognostic BC gene score, was calculated from the average z-scores of the selected upregulated DEGs, emphasizing their association with improved survival. In all three independent datasets, analysis using the Kaplan−Meier method and Cox proportional hazards models revealed that high BC-GS groups had significantly better OS than the low BC-GS groups (HR = 0.49, 95% CI = 0.29–0.84, *p* = 0.008 in GSE176307; HR = 0.70, 95% CI = 0.49–0.99, *p* = 0.043 in IMvigor210; HR = 0.36, 95% CI = 0.20–0.67, *p* = 0.00081 in UC-GENOME) (Fig. 2a).

**Fig. 3:**
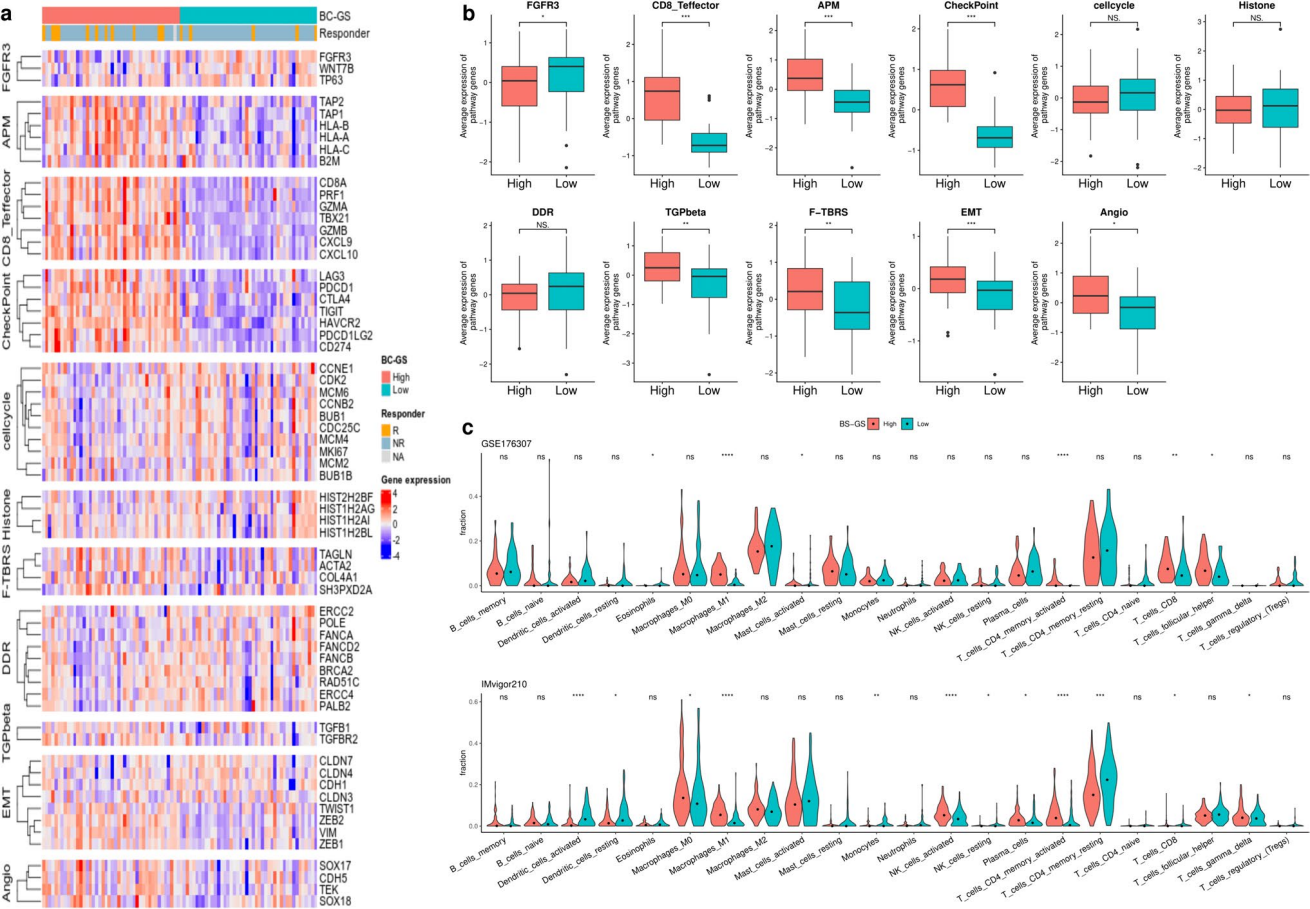
Core biological pathways and cell dynamics according to the BC-GS subgroups. **a** Relationship between the BC-GS and core biological pathways in the GSE176307 dataset. The rows of the heat map are sorted according to the gene expression of pathways (Z scores). **b** Box plots displaying the average levels of gene expression for the core pathways in high and low groups in the BC-GS. p values for the two-sided Welch tests are displayed for the selected comparison. **c** Violin plot displaying CIBERSORT results for GSE176307 and IMvigor210. This violin plot shows the cell fractions inferred by CIBERSORT for the different patient groups categorized according to the BC-GS. The dot represents the median. For symbols denoting statistical significance, the following conventions were followed: ns, *p* > 0.05; *, *p* ≤ 0.05; **, *p* ≤ 0.01; ***, *p* ≤ 0.001

Initially, we analyzed the 10 genes comprising the BC-GS to assess their contributions to the differences observed in OS. Enrichment and subsequent gene network analyses revealed that the plasma lipoprotein and complement cascade pathways were prevalent, with significant co-expression and physical interactions among the involved genes (Fig. 2b, c). Significant differences in gene expression were observed in most cancer-related biological pathways between the high and low BC-GS groups. Notably, differences in immune-related pathways, in particular in the CD8^+^ T effector, APM, and checkpoint pathways, were evident (Fig. 3a, b). Analysis using the CIBERSORT method revealed the distinct distribution of cell types between the high and low BC-GS groups across different datasets. In particular, patients with high BC-GS exhibited a higher proportion of “T cell CD4^+^ memory activated” and “M1 macrophage” cells (Fig. 3c).

### Investigating the clinical and biological significance of the combination of BC-GS and TMB

**Fig. 4:**
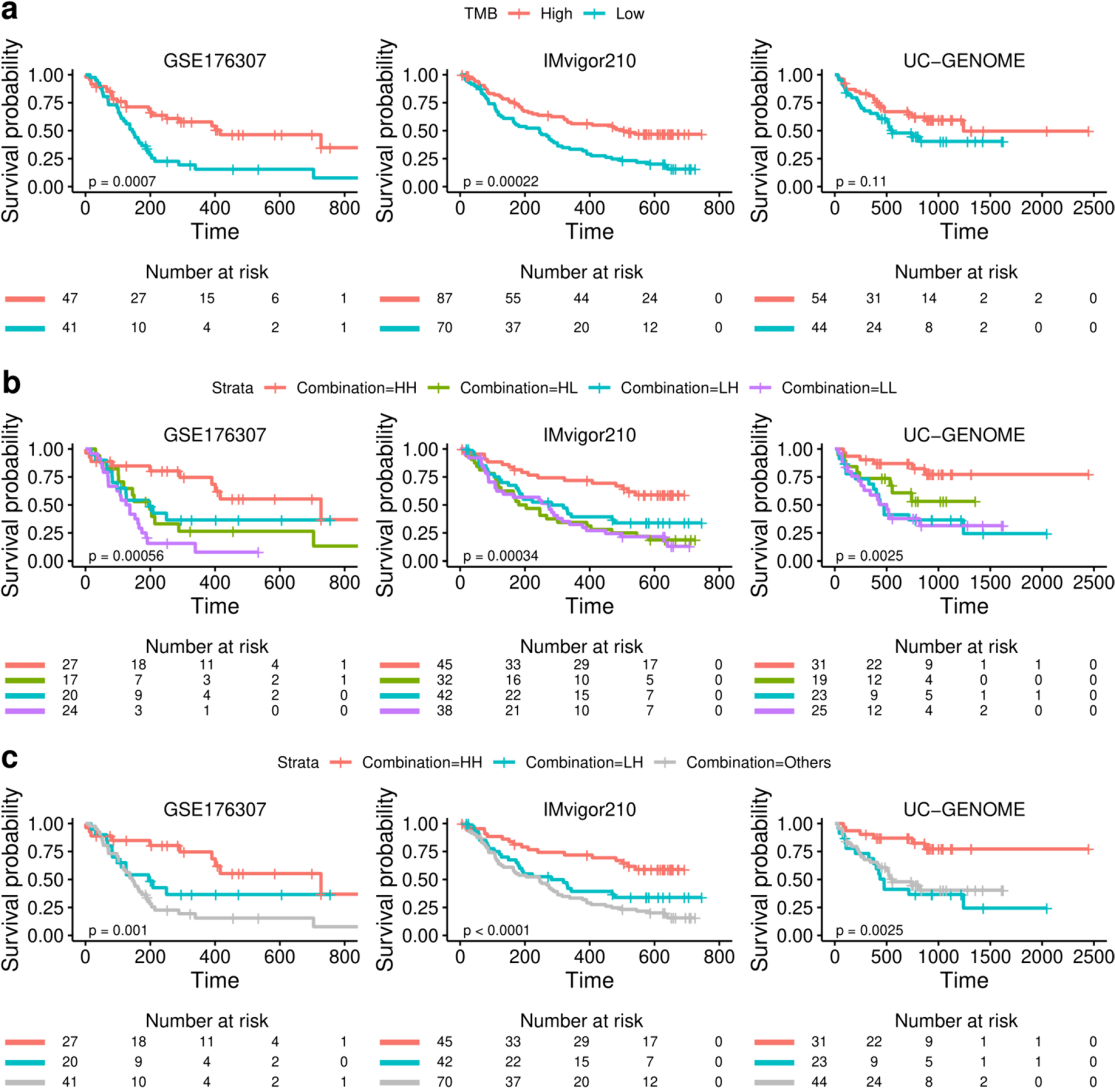
Survival plots according to the combination of the BC-GS and TMB. The OS curves display the impact of tumor mutational burden (TMB) a, the combination of BC-GS and TMB b, and HH versus LH and others c on patient survival across three independent datasets (GSE176307, IMvigor210, and UC-GENOME). Tick marks on the survival curves represent censoring events. The p-value was calculated using the two-tailed log-rank-sum test and used to determine the statistical significance of survival differences between groups. HH: high in both BC-GS and TMB; HL, high in BC-GS but low in TMB; LH, low in BC-GS but high in TMB; LL: low in both BC-GS and TMB

The TMB, recognized as a significant biomarker for antitumor response to ICI across multiple cancer types, exhibited statistically significant prognostic differences in two of the three independent datasets (Supplementary Fig. 8). The high TMB groups had significantly better OS than the low TMB groups (HR = 0.40, 95% CI = 0.23–0.69, *p* = 0.0007 in GSE176307; HR = 0.48, 95% CI = 0.32–0.71, *p* = 0.0002 in IMvigor210; HR = 0.62, 95% CI = 0.35–1.12, *p* = 0.11 in UC-GENOME) (Fig. 4a).

To assess the combined potential of the BC-GS and TMB, patients were stratified into high and low categories based on the median values for both metrics. Survival outcomes were analyzed across four subgroups: high BC-GS and high TMB (HH), high BC-GS and low TMB (HL), low BC-GS and high TMB (LH), and low BC-GS and low TMB (LL). Significant differences in OS were observed among the four subgroups across all datasets (*p* = 0.0006, 0.0003, and 0.0025 for GSE176307, IMvigor210, and UC-GENOME, respectively) (Fig. 4b). Importantly, significant differences in OS were observed between HH and LH patients upon reclassifying the high TMB group using BC-GS (*p* = 0.001, < 0.0001, and 0.0025 in GSE176307, IMvigor210, and UC-GENOME, respectively) (Fig. 4c). This is indicative of significant heterogeneity within the high TMB group. A similar pattern was observed between the HH and HL groups within the high BC-GS group (Supplemental Fig. 9).

**Fig. 5:**
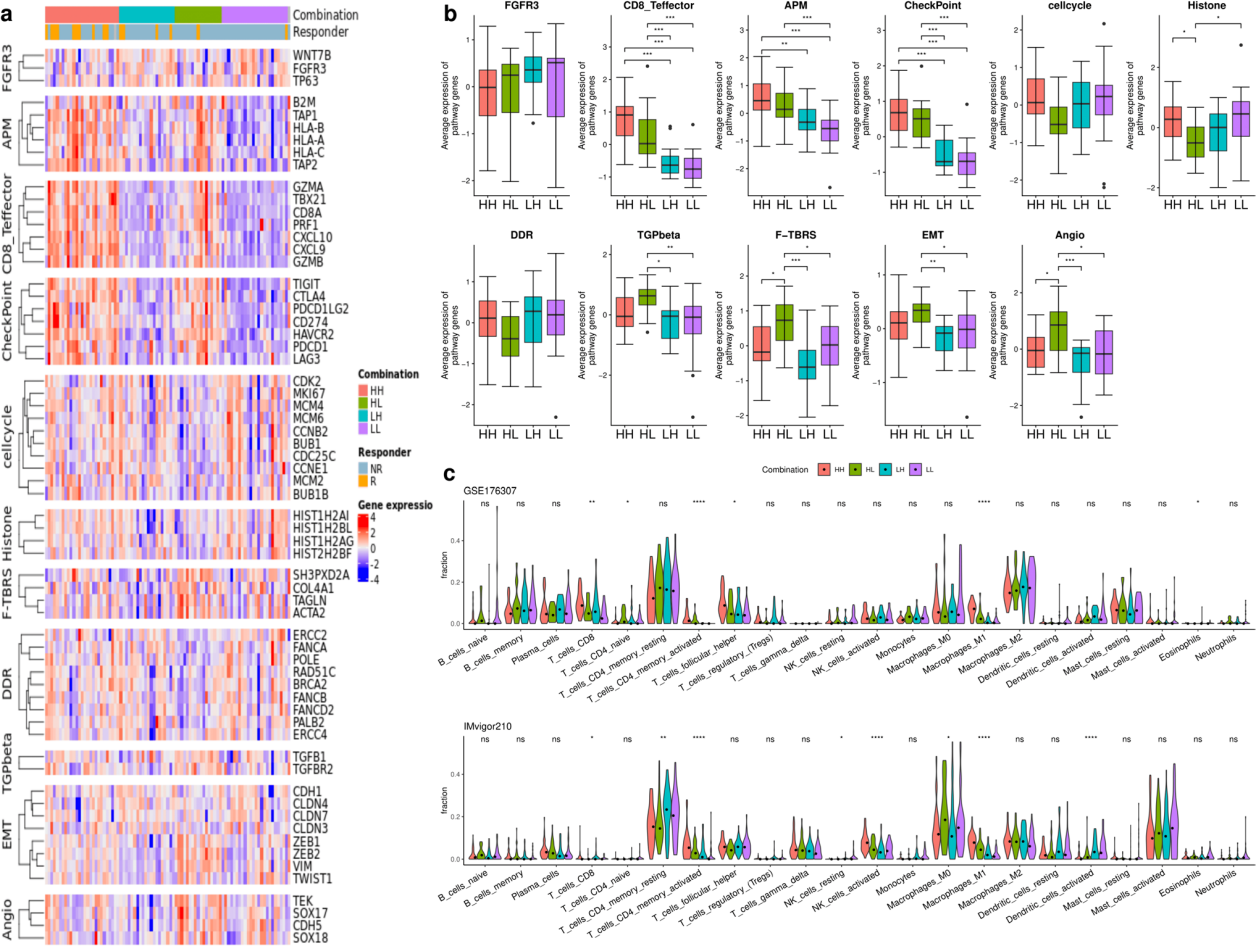
Core biological pathways and cell dynamics according to the combination of BC-GS and TMB. **a** Relationship between a combination of BC-GS and TMB and core biological pathways in the GSE176307 dataset. The rows of the heat map were sorted according to pathway gene expression (Z-scores). **b** Box plots displaying the average levels of gene expression for the core pathways in the high and low groups as determined using a combination of BC-GS and TMB. **c** The violin plot shows the cell fractions inferred by CIBERSORT for the different patient groups categorized according to a combination of BC-GS and TMB. Dots represent median values. The p values presented were derived using analysis of variance (ANOVA) followed by a post hoc Tukey's honest significant difference test. For symbols denoting statistical significance, the following conventions were used: ns, *p* > 0.05; *, *p* ≤ 0.05; **, *p* ≤ 0.01; ***, *p* ≤ 0.001. HH: high in both BC-GS and TMB; HL, high in BC-GS but low in TMB; LH, low in BC-GS but high in TMB; LL: low in both BC-GS and TMB

After analyzing 11 core biological pathways associated with cancer, a distinct linear trend in gene expression levels was observed within immune-related pathways, specifically the CD8^+^ T effector, APM, and checkpoint pathways. This trend followed a significant order (*p* < 0.001) among the four groups: HH, HL, LH, and LL (Fig. 5a, b, Supplementary Table 5). Gene expression levels in the CD8^+^ T effector, APM, and checkpoint pathways were significantly higher in the HH groups than in the LH groups (*p* < 0.001) (Fig. 5b). Conversely, in the F-TBRS (TGF-*β* response signature) and angiogenesis pathways, the gene expression levels in the HH groups were lower than those in the HL groups (*p* = 0.0293 and 0.0235) (Fig. 5b; Supplementary Fig. 10). Consistent with the observations of pathway analysis, the results of the CIBERSORT analysis revealed a distinct linear progression in the proportions of “T cell CD4^+^ memory activated” and “macrophages M1” across the HH, HL, LH, and LL groups (*p* < 0.001 in GSE176307 and IMvigor210, respectively).

## Discussion

In this study, we identified the cell populations associated with improved OS in patients with BC receiving ICI therapy using an integrated analysis of scRNA-seq and bulk RNA-seq data. This comprehensive analysis enabled us to identify DEGs within these critical cells, leading to the development of a BC-GS biomarker that effectively categorized patients with BC into groups with high or low risks of survival after ICI treatment. The consistency in the performance of the BC-GS in independent datasets, including IMvigor210 and UC-genome, further validated this classification. Therefore, our findings can significantly improve the prediction of the therapeutic response of patients with BC to ICI treatment.

Building on the results of BC-GS, we further investigated the immunological mechanisms using CIBERSORT. Our findings indicated that a high proportion of macrophages was associated with improved survival, suggesting that macrophages play a key role in the TME of patients with BC treated with ICI. Recent advancements in single-cell omics techniques have led to the identification of distinct subpopulations of tumor-associated macrophages (TAMs), including a subset known as lipid-associated TAMs (LA-TAMs) [32–34]. Timperi and colleagues have identified two specific LA-TAM subpopulations within breast cancer: LAM-APOC1, known for its unique origin, and monocyte-derived LAM-STAB1 [35]. LAM-APOC1 is particularly important because of its immune activity, as indicated by its association with cytotoxic/experienced CD8^+^ T cells and regulatory T cells (Tregs). Our findings revealed that the LA-TAM signature genes, such as *APOE, C1QB, C1QA, APOC1*, and *C1QC*, were expressed significantly in M1/M2 macrophages compared to that in monocytes, as depicted in Fig. 1a, b, f, and Supplementary Fig. 7. This suggests that the Scissor^⁻^ cells, associated with improved survival in our study, represented a subtype of LA-TAMs, possibly corresponding to LAM-APOC1.

TMB is widely used as an immunotherapeutic marker of antitumor activities [36]. Recently, several studies have analyzed its relationship with survival outcomes [37, 38]. Our study not only assessed the effectiveness of the BC-GS as a survival marker but also investigated the role of TMB in predicting survival outcomes. We found that both markers could be used independently and significantly to predict the treatment outcomes. Furthermore, combining the BC-GS with TMB enhanced the precision of survival prediction across BC patient cohorts. This combined approach enabled more detailed patient stratification post-immunotherapy, as the BC-GS could effectively classify heterogeneity within high TMB groups. Interestingly, while the BC-GS and TMB aligned in terms of immune pathways, such as CD8^+^ T effector functions, they diverged in terms of metastasis pathways, including that involving TGF-*β*, indicating complementary effects on tumor biology. Thus, the combination of BC-GS and TMB may be used for predicting OS owing to their distinct effects on immune activation and tumor progression.

Our study has several limitations. First, the retrospective design posed challenges, particularly in harmonizing data across disparate datasets. Additionally, the use of simple median cutoffs for BC-GS and TMB, while practical, may lack the precision that a more systematic approach could provide. Furthermore, there is a need to compare these findings with well-established biomarkers, such as PD-L1 levels measured by immunohistochemistry (IHC), to gain more accurate and deeper insights. These issues, along with the variability in clinical variables, highlight the necessity of a prospective study that integrates diverse data, including clinical, radiological, and pathological information with PD-L1 IHC, to ensure a more comprehensive and precise validation of BC-GS. Nevertheless, despite these limitations, BC-GS consistently demonstrated significant survival differences across diverse datasets. This robustness underscores its potential as a reliable and generalizable biomarker for BC, capable of offering valuable prognostic insights even in the absence of detailed clinical data. Consequently, BC-GS holds promise as a key candidate for broader clinical application, warranting further validation in prospective studies.

In conclusion, we integrated the data from scRNA-seq and bulk RNA-seq to establish the BC-GS score and demonstrated the efficacy of the BC-GS across diverse BC patient cohorts for predicting the survival outcomes of palliative ICI treatment. Furthermore, the use of the BC-GS-TMB combination can improve patient stratification and guide personalized treatment. However, further validation in clinical trials is necessary to confirm the clinical utility and robustness of this method.

## Supplementary Information

Below is the link to the electronic supplementary material.

Supplementary file1 (PDF 1112 KB)

## Data Availability

The IMvigor210 package can be accessed at http://research-pub.gene.com/IMvigor210CoreBiologies/packageVersions/IMvigor210CoreBiologies_1.0.0.tar.gz. The custom code used to identify ICI-related gene signatures in patients with BC is available at the GitHub repository (https://github.com/mykamki/finding-immunotherapeutic-marker).

## Abbreviations

BC: Bladder cancer
BC-GS: Bladder cancer gene signature
DEGs: Differentially expressed genes
ICI: Immune checkpoint inhibitors
TMB: Tumor mutation burden
TME: Tumor microenvironment
