# Supplementary material for: Integration of bulk and single-cell RNA-seq reveals prognostic gene signatures in patients with bladder cancer treated with immune checkpoint inhibitors
Source: Cancer Immunol Immunother. 2024 Dec 21;74(1):28. doi: 10.1007/s00262-024-03839-7 (PMC11663206; doi:10.1007/s00262-024-03839-7)
Supplement: Supplementary file 1 — Supplementary file1 (PDF 1112 KB) [file 262_2024_3839_MOESM1_ESM.pdf]

Supplementary Information

Supplementary Table 1. Dataset analyzed in this study

GEO Accession	Tissue	Platform	Number of cases	PMID	Level of data	Source
GSE135337	Primary tumor samples	Illumina NovaSeq 6000	N = 7	34480339	Read count	https://www.ncbi.nlm.nih.gov/geo/download/?acc=GSE135337&format=file
GSE176307	FFPE tumor tissue from a primary or metastatic site	Ion Torrent S5 XL	N = 89	34294892	TPM quantified using the RSEM	https://www.ncbi.nlm.nih.gov/geo/download/?acc=GSE176307&format=file&file=GSE176307%5FBACI%5Ftpm%5Fgene%2Ematrix%2Etsv%2Egz
IMvigor210	FFPE tumor tissue	Illumina TruSeq RNA	N = 348	29443960	TPM	http://research-pub.gene.com/IMvigor210CoreBiologies/packageVersions/IMvigor210CoreBiologies_1.0.0.tar.gz.
UC-GENOME	FFPE tumor tissue	Illumina TruSeq RiboZero Gold protocol	N=180	36333289	mRNA expression UQ_Log2_zscore	http://www.cbioportal.org/

Supplementary Table 2. Detailed specific parameters of quality control (QC) in scRNA-seq dataset (GSE135337)

	Default (SCISSOR)	BC1	BC2	BC3	BC4	BC5	BC6	BC7
QC and selecting cells for further analysis								
Minimum number of cells containing at least the detected feature	400	3	3	3	3	3	3	3
Minimal number of features	0	200	1000	500	700	500	500	400
Maximal number of features	max	7500	6000	6000	3000	4000	5000	4000
Percent of mitochondrial feature	max	10	10	10	10	10	7.5	10
Cluster the cells								
Number of components	10	10	10	10	10	15	10	10
Resolution	0.6	1.6	0.5	1.6	0.5	2	4	2
		**BC1**	**BC2**	**BC3**	**BC4**	**BC5**	**BC6**	**BC7**
Cells after QC (sup)		3195	4948	3046	5118	8630	6161	5521
No. cell before QC (original dataset from GEO database)		3218	5006	3100	5131	8643	6167	5523
No. cell after QC		3193	4948	3073	5023	8585	6095	5383
No. cluster		17	9	20	7	25	43	24

Supplementary Table 3. List of gene markers used for cluster annotation

Cell name	Marker gene	PMID
Myeloid/macrophage	LYZ	33033240
T cell	CD3D, CD2, CD3E	33033240, 32553173, 33033240
Endothelial cells	VWF	34385456
B cell	CD79A, MZB1	33033240, 28654764
Fibroblasts	TAGLN	31462402
Epithelial cells	KRT18	31462402

Supplementary Table 4. Gene sets of core biological pathways

Gene signature	Gene symbols
FGFR3	FGFR3, TP63, WNT7B
CD8 T effector	CD8A, CXCL10, CXCL9, GZMA, GZMB, PRF1, TBX21
APM	TAP1, TAP2, B2M, HLA-A, HLA-B, HLA-C
Immune checkpoint	CD274, PDCD1LG2, CTLA4, PDCD1, LAG3, HAVCR2, TIGIT
Cell cycle	MKI67, CCNE1, BUB1, BUB1B, CCNB2, CDC25C, CDK2, MCM4, MCM6, MCM2
Histone	HIST1H2AI, HIST1H2AG, HIST1H2BL, HIST2H2BF
DDR	BRCA2, ERCC2, ERCC4, FANCA, FANCB, FANCD2, PALB2, POLE, RAD51C
TGF-ß	GTGFB1, TGFBR2
Pan-F-TBRS	ACTA2, COL4A1, TAGLN, SH3PXD2A
EMT	CLDN3, CLDN7, CLDN4, CDH1, VIM, TWIST1, ZEB1, ZEB2
Angiogenesis	TEK, CDH5, SOX17, SOX18

Abbreviations: antigen-presenting mechanism (APM), DNA damage response (DDR), epithelial-mesenchymal transition (EMT).

Supplementary Table 5. Linearity of gene expression of core biological pathways according to the combination of BC-GS and TMB

MainKey	SubKey	Estimate	StdError	TValue	PValue
FGFR3	HH->HL->LH->LL	0.1030387	0.0747068	1.3792422	0.1713964
CD8 T effector	HH->HL->LH->LL	-0.534814	0.0569955	-9.383453	8.15E-15
APM	HH->HL->LH->LL	-0.393624	0.062572	-6.290747	1.27E-08
Immune checkpoint	HH->HL->LH->LL	-0.495918	0.0500489	-9.908656	6.98E-16
Cell cycle	HH->HL->LH->LL	0.0187431	0.0738699	0.2537318	0.8003084
Histone	HH->HL->LH->LL	0.0152059	0.0752382	0.2021029	0.8403136
DDR	HH->HL->LH->LL	0.0435983	0.0679714	0.6414221	0.5229543
TGF-ß	HH->HL->LH->LL	-0.175034	0.0695809	-2.515544	0.0137435
Pan-F-TBRS	HH->HL->LH->LL	-0.111846	0.0710671	-1.573802	0.1192051
EMT	HH->HL->LH->LL	-0.080327	0.0389784	-2.060809	0.0423424
Angiogenesis	HH->HL->LH->LL	-0.10203	0.0755842	-1.349886	0.1805949

Abbreviations: antigen-presenting mechanism (APM), DNA damage response (DDR), epithelial-mesenchymal transition (EMT).

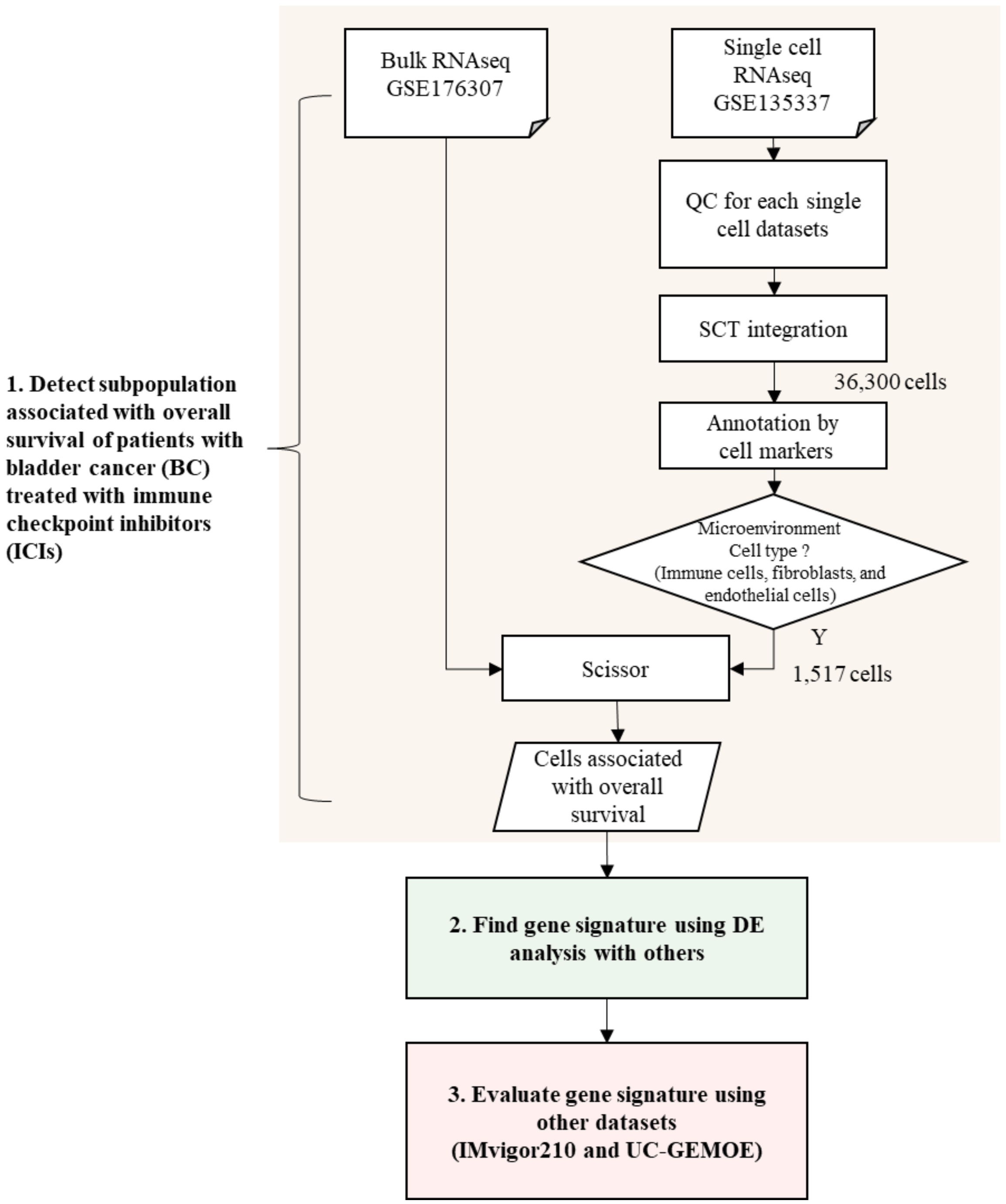

Supplementary Figure 1. Workflow of this study.

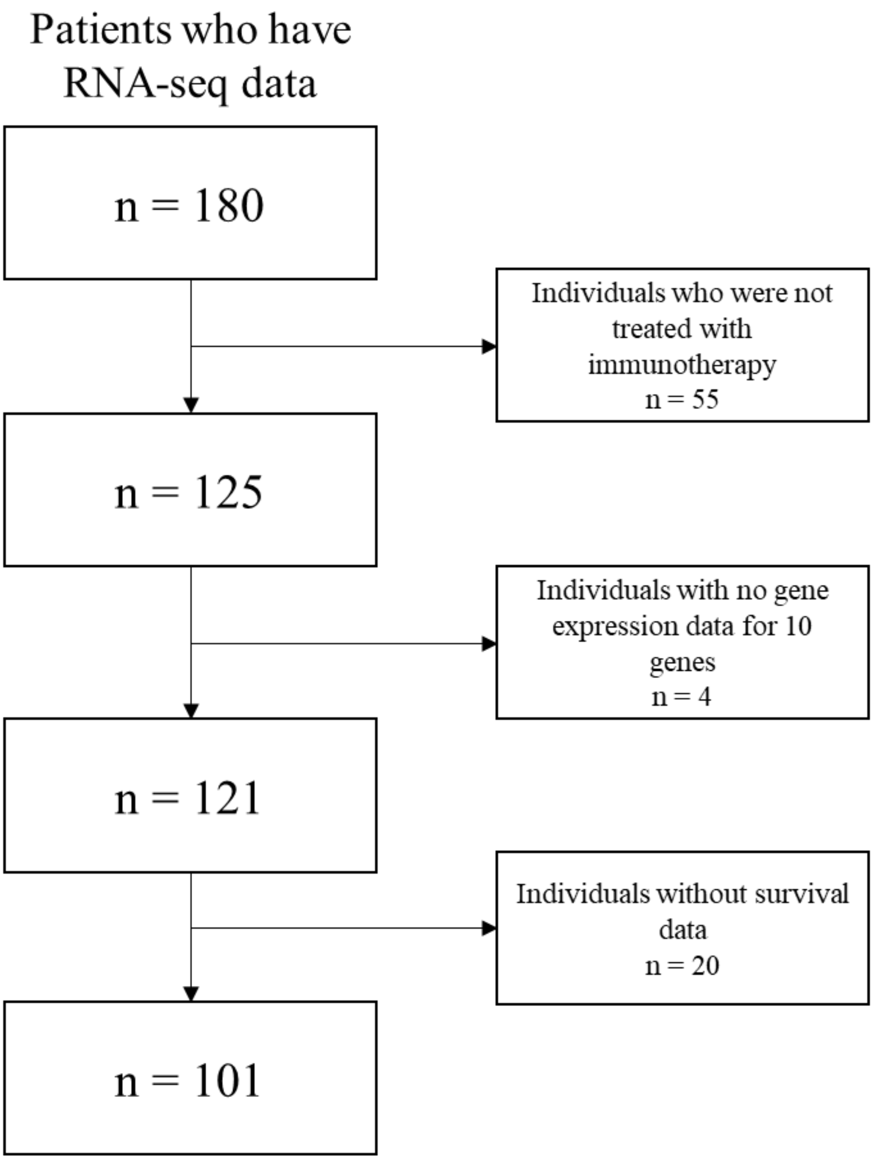

Supplementary Figure 2. Flow chart showing patient selection in UC-GENOME.

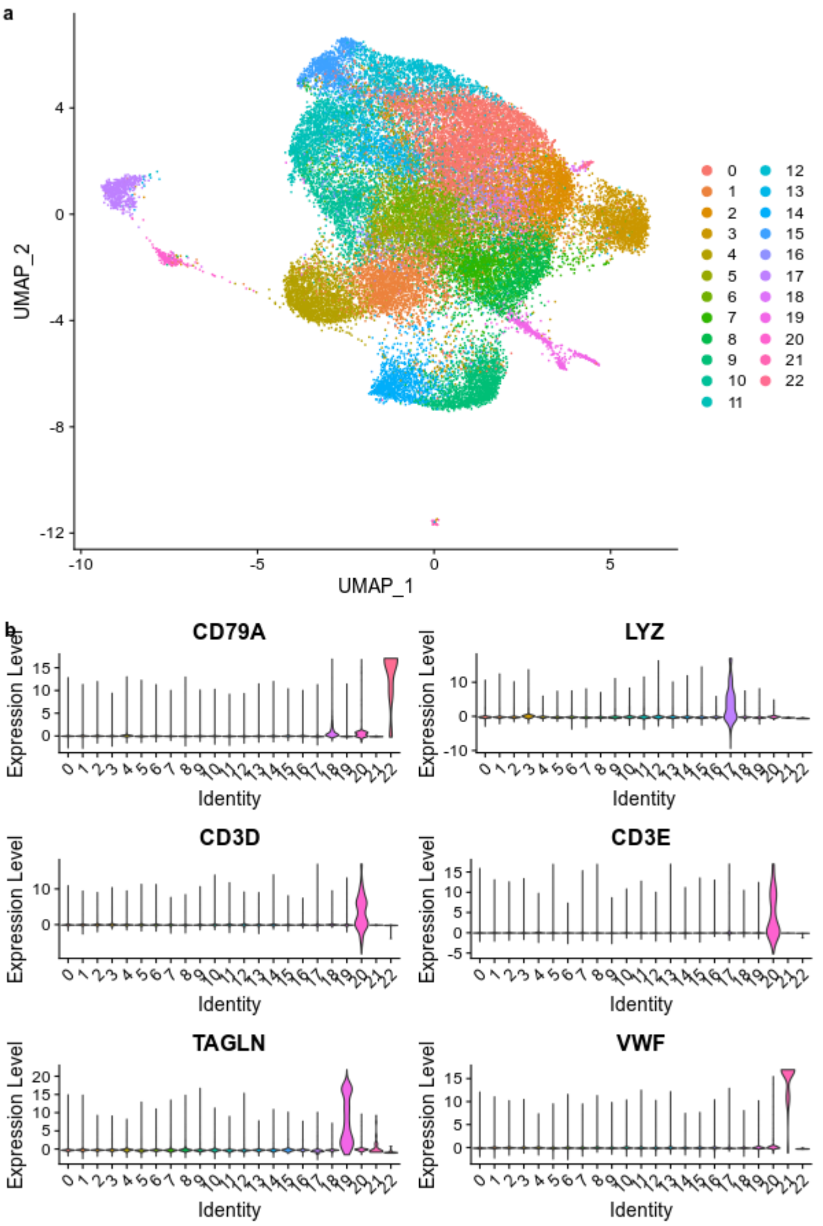

Supplementary Figure 3. Integrated scRNA-seq data from seven patients in GSE135337
a. Uniform Manifold Approximation and Projection (UMAP) visualization of 36,300 bladder cancer cells integrated via single cell transformation. b. According to marker genes used in Lai et al. 2021, the following cell types were identified in different clusters; Cluster 17: myeloid/macrophages (LYZ marker); Cluster 22: B cells (CD79A marker); Cluster 20: T cells (CD3D, CD3E markers); Cluster 19: fibroblasts (TAGLN marker); Cluster 21: endothelial cells (VWF marker).

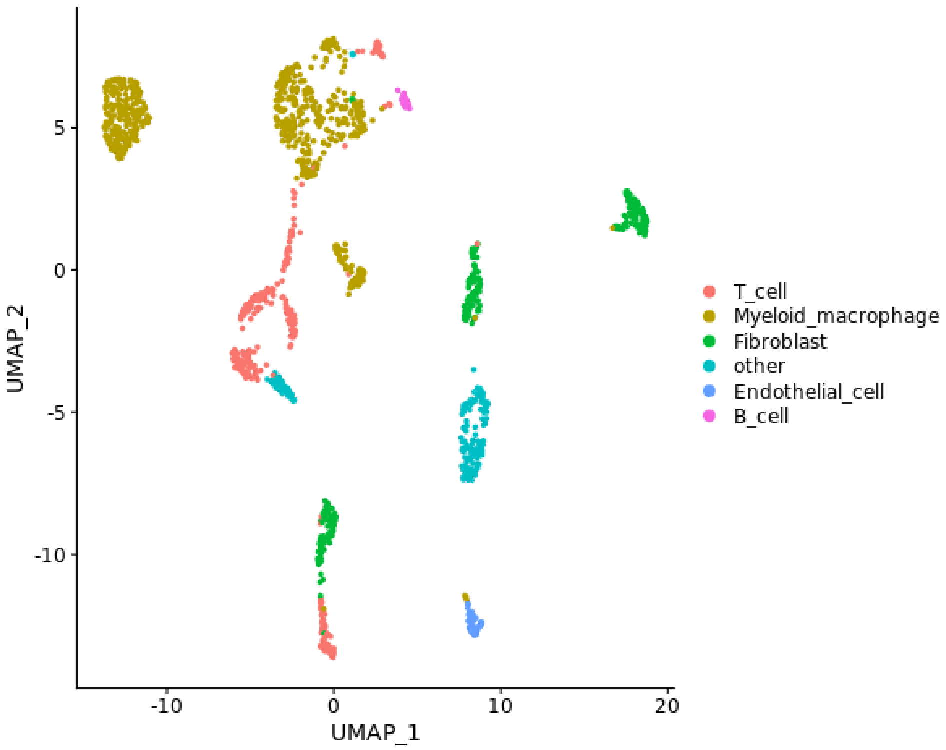

Supplementary Figure 4. UMAP visualization of 1,517 cells in bladder cancer, except for epithelial cells

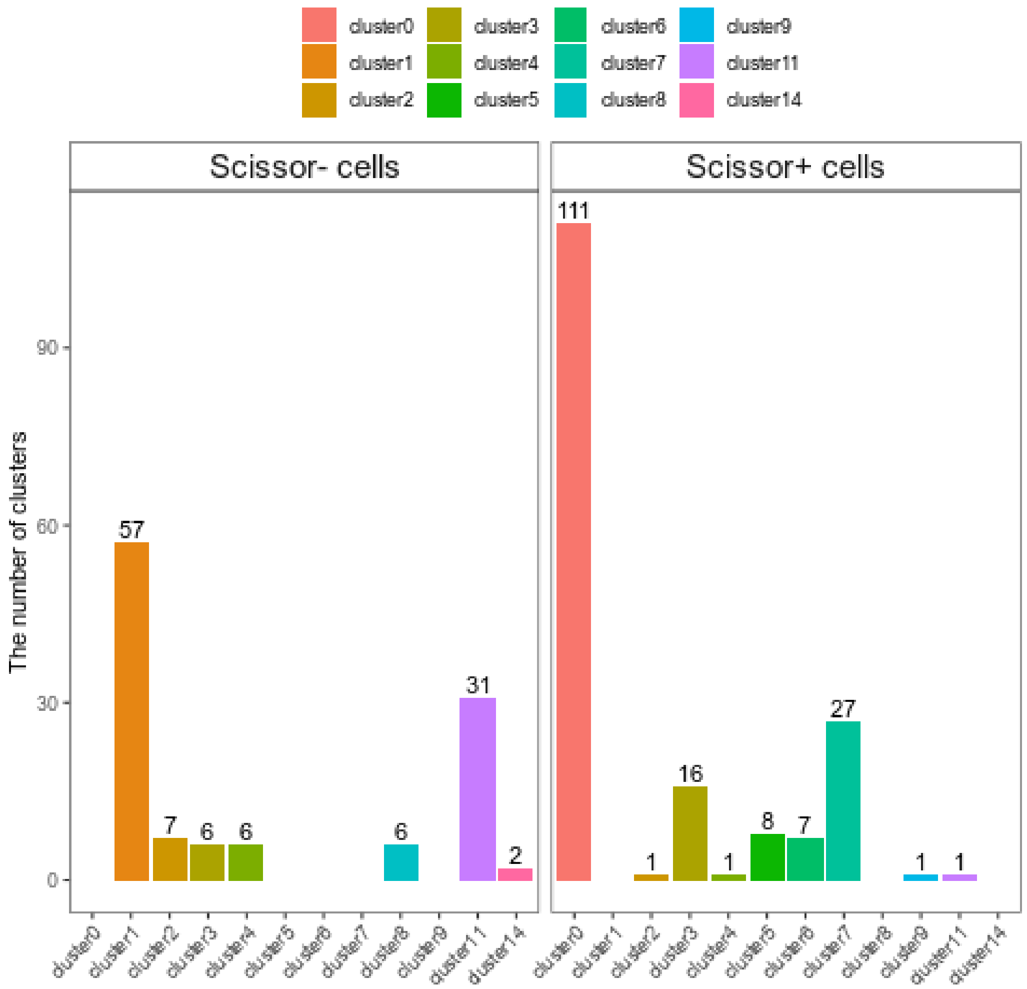

Supplementary Figure 5. Distribution of clusters in Scissor$^-$ and Scissor$^+$ cells

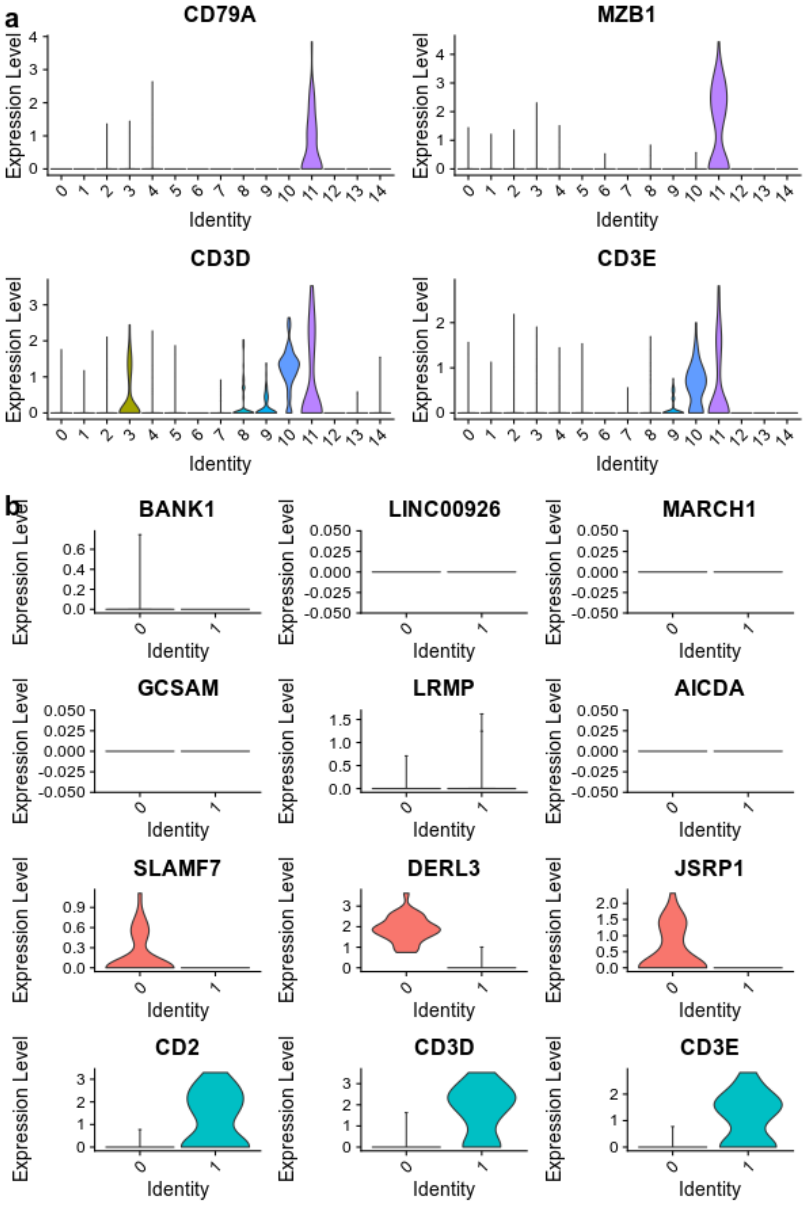

Supplementary Figure 6. Additional cluster of cluster 11

a. Cluster 11 was expressed in both B cell (CD79A and MZB1) and T cell (CD3D and CD3E) marker genes. b. Approximately 60 clusters and 11 cells were re-clustered and classified into two clusters; Cluster 0: plasma cells (SLAMF7, DERL3, and JSRP1markers); Cluster 1: T cells (CD2, CD3D, and CD3E markers).

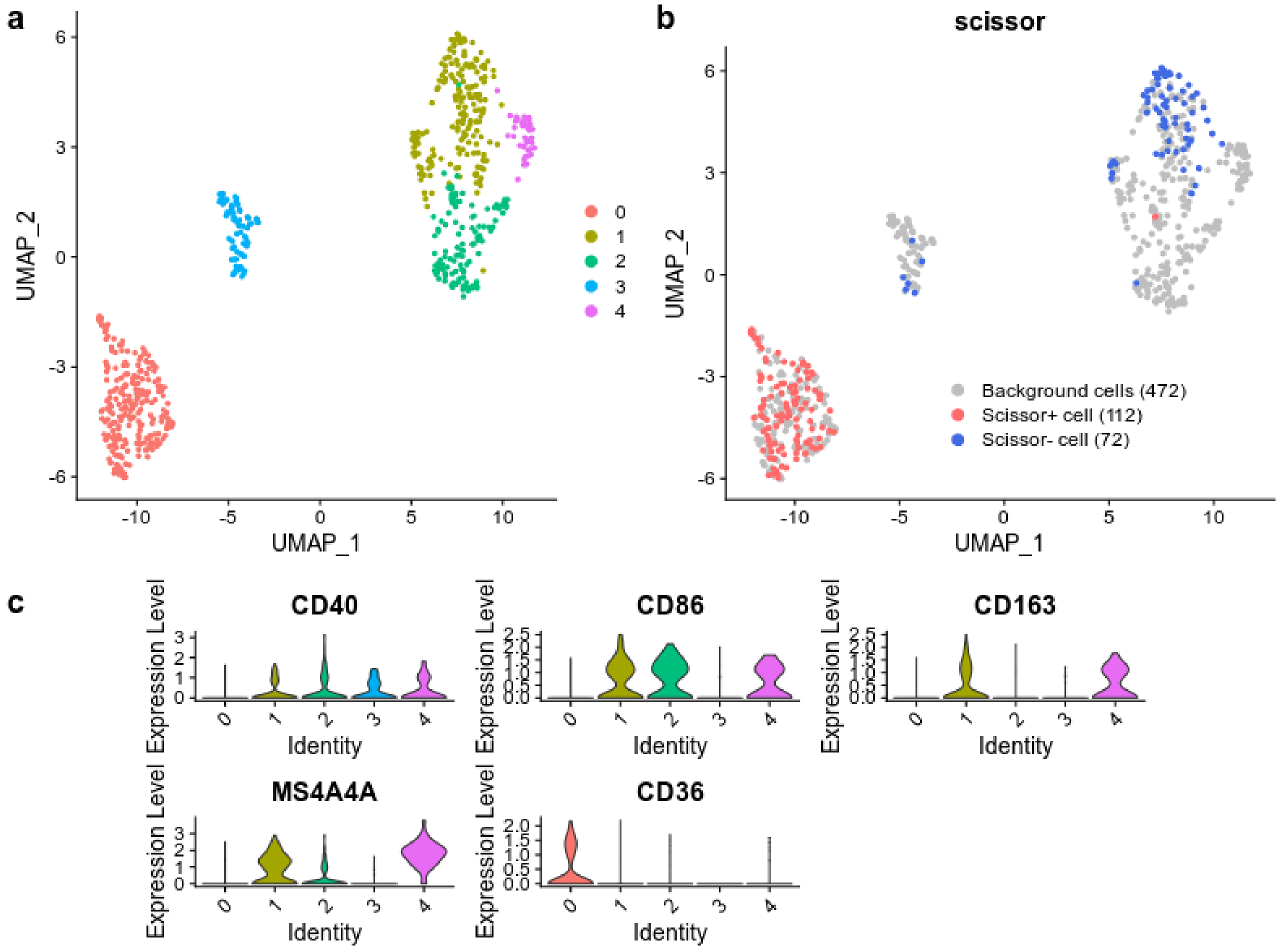

Supplementary Figure 7. Re-clustering of 656 myeloid/macrophage cells

a. UMAP visualization of 656 re-clustered myeloid/macrophage cells. Five clusters were divided into three groups; group 1: cluster 0; group 2: cluster 3; group 3: cluster 1, 2, and 4. b. UMAP visualization of Scissor^{-} and Scissor^{+} cells among 656 myeloid/macrophage cells. c. The violin plot of marker genes in 656 re-clustered myeloid/macrophage cells. CD40 and CD86 represent M1 macrophages, CD163 and MS4A4A represent M2 macrophages, and CD36 represents monocytes.

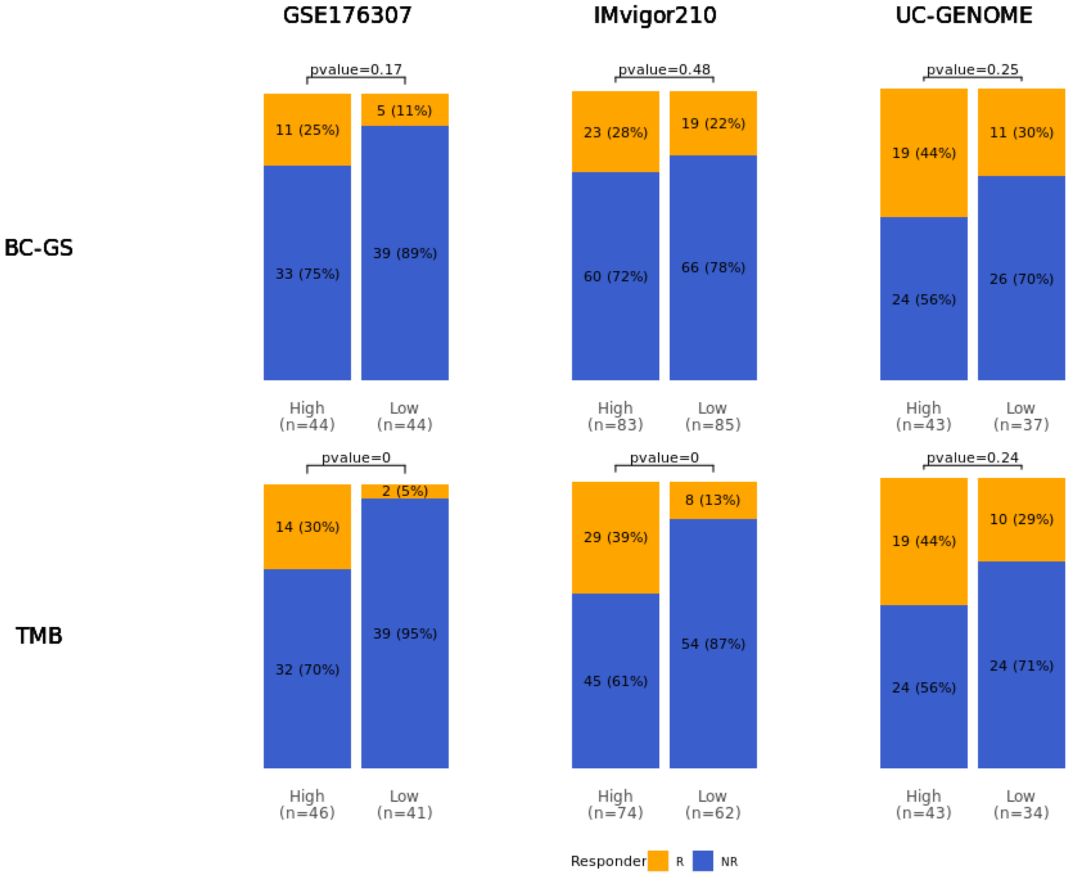

Supplementary Figure 8. Tumor response rate to ICIs according to the BC-GS and TMB
The distribution of ICI responders stratified according to the BC-GS and TMB was examined in three independent datasets (GSE176307, IMvigor210, and UC-GENOME). The responders were those who exhibited a complete response (CR) or partial response (PR). Categorical variables were compared using Fisher's exact test or the chi-squared test. The *p* values were expressed by rounding off to two decimal places. R: responders; NR: non-responders.

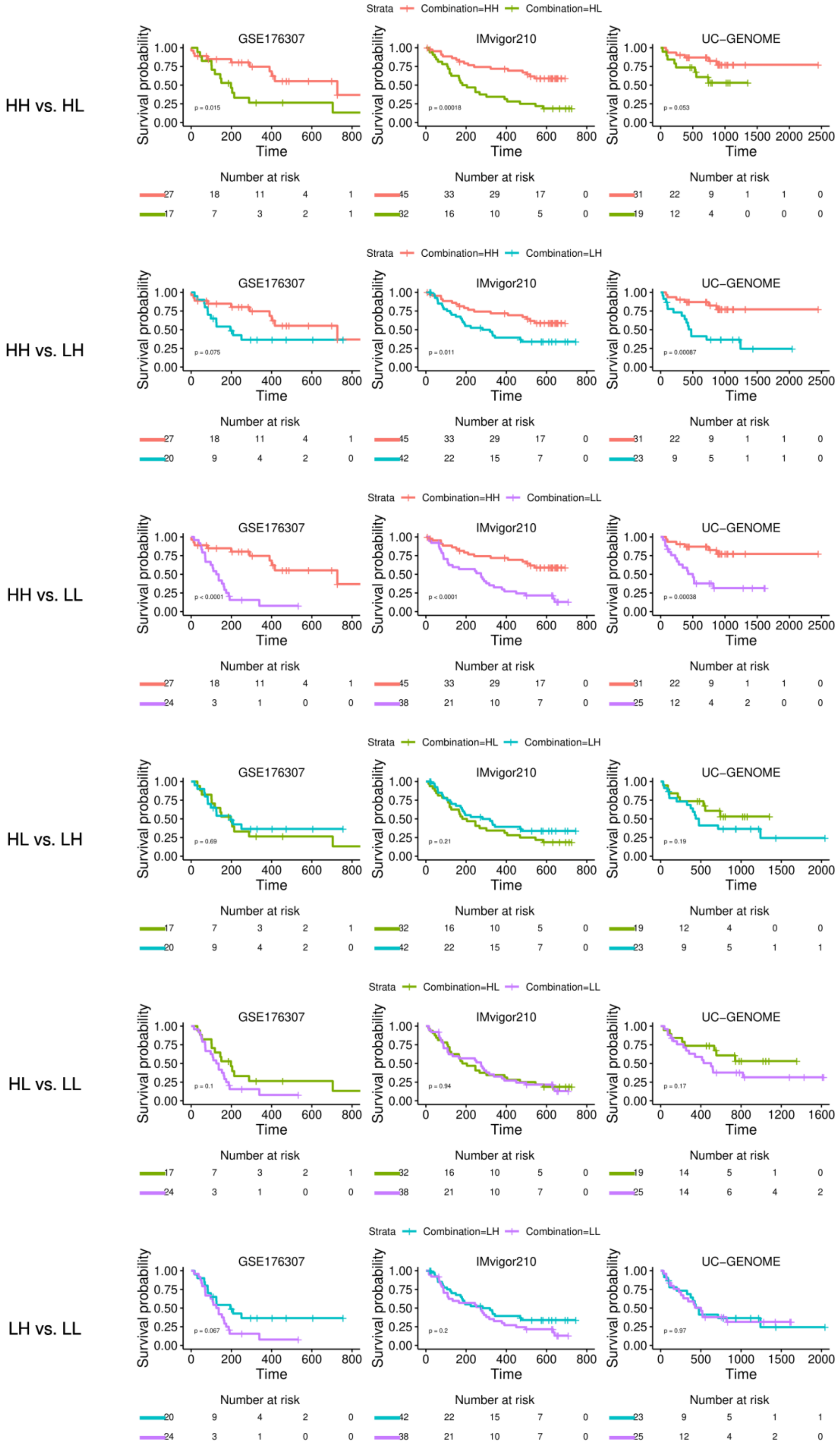
HH vs. HL
Strata Combination=HH Combination=HL
GSE176307
IMvigor210
UC−GENOME
p = 0.015
p = 0.00018
p = 0.053
HH vs. LH
Strata Combination=HH Combination=LH
p = 0.075
p = 0.011
p = 0.00087
HH vs. LL
Strata Combination=HH Combination=LL
p < 0.0001
p < 0.0001
p = 0.00038
HL vs. LH
Strata Combination=HL Combination=LH
p = 0.69
p = 0.21
p = 0.19
HL vs. LL
Strata Combination=HL Combination=LL
p = 0.1
p = 0.94
p = 0.17
LH vs. LL
Strata Combination=LH Combination=LL
p = 0.067
p = 0.2
p = 0.97
Survival probability
Time
Number at risk

Supplementary Figure 9. Overall survival (OS) plots according to the combination of BC-GS and TMB

The OS plots for the combination of BC-GS and TMB were analyzed in three independent datasets (GSE176307, IMvigor210, and UC-GENOME). HH: high in both BC-GS and TMB; HL, high in BC-GS but low in TMB; LH, low in BC-GS but high in TMB; LL: low in both BC-GS and TMB.

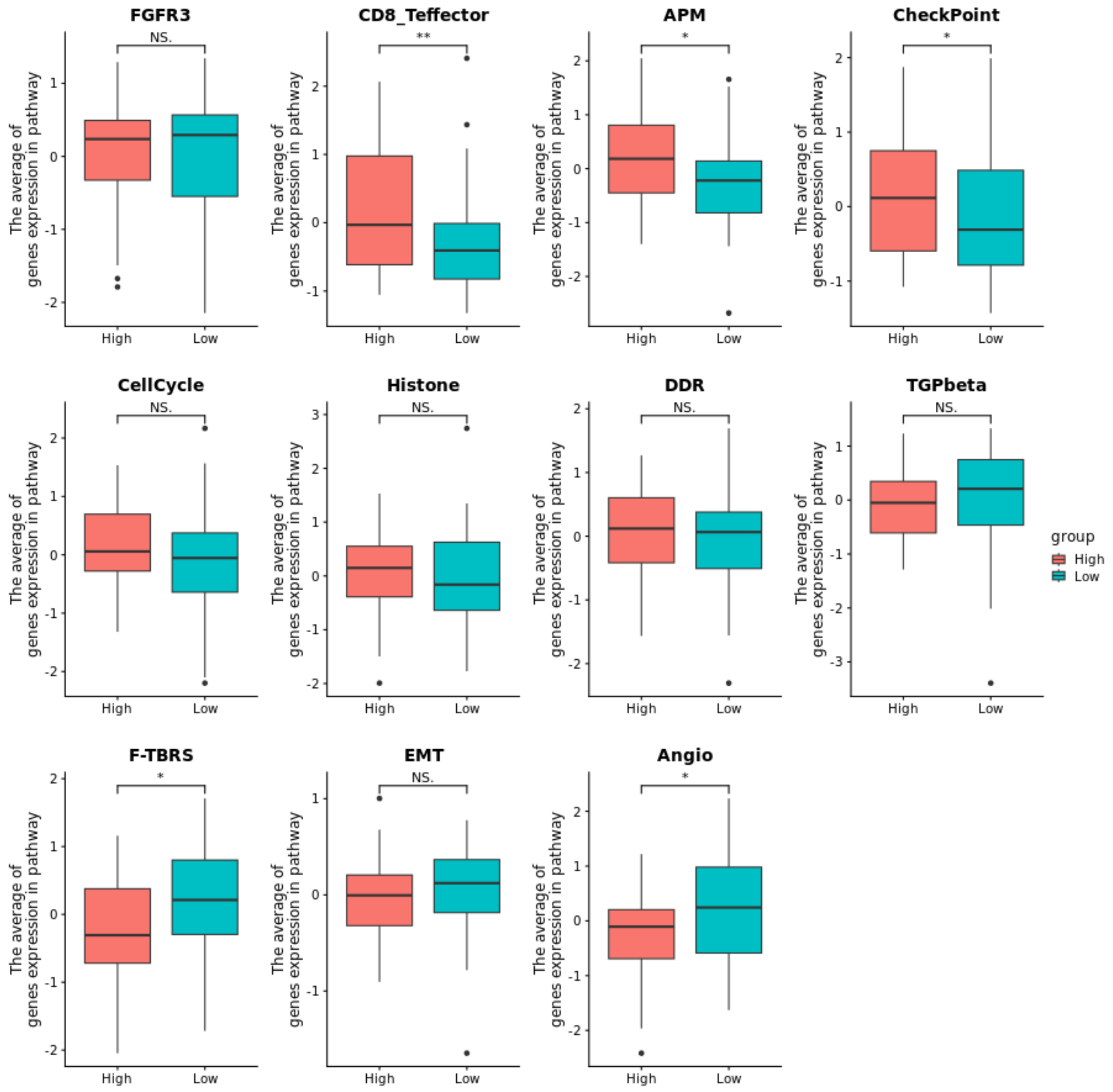

Supplementary Figure 10. Box plots displaying gene expression of core biological pathways according to TMB. Box plots displaying the average levels of gene expression for the core pathways in high and low groups according to TMB. *p* values for two-sided Welch's tests are displayed for the selected comparison. For symbols denoting statistical significance, the following conventions were used: ns, $p > 0.05$; *, $p \leq 0.05$; **, $p \leq 0.01$; ***, $p \leq 0.001$.
